# Supplementary material for: Mortality of individuals with antemortem genetic testing for PRNP variants in the United States, 1998–2024
Source: medRxiv. 2025 Oct 9:2025.10.03.25337271. Preprint. [Version 2] doi: 10.1101/2025.10.03.25337271 (PMC12622085; doi:10.1101/2025.10.03.25337271)
Supplement: Supplement 3 [file NIHPP2025.10.03.25337271v2-supplement-3.pdf]

medRxiv preprint doi: https://doi.org/10.1101/2025.10.03.25337271; this version posted October 9, 2025. The copyright holder for this preprint
(which was not certified by peer review) is the author/funder, who has granted medRxiv a license to display the preprint in perpetuity.
It is made available under a CC-BY 4.0 International license .

Supplementary Materials

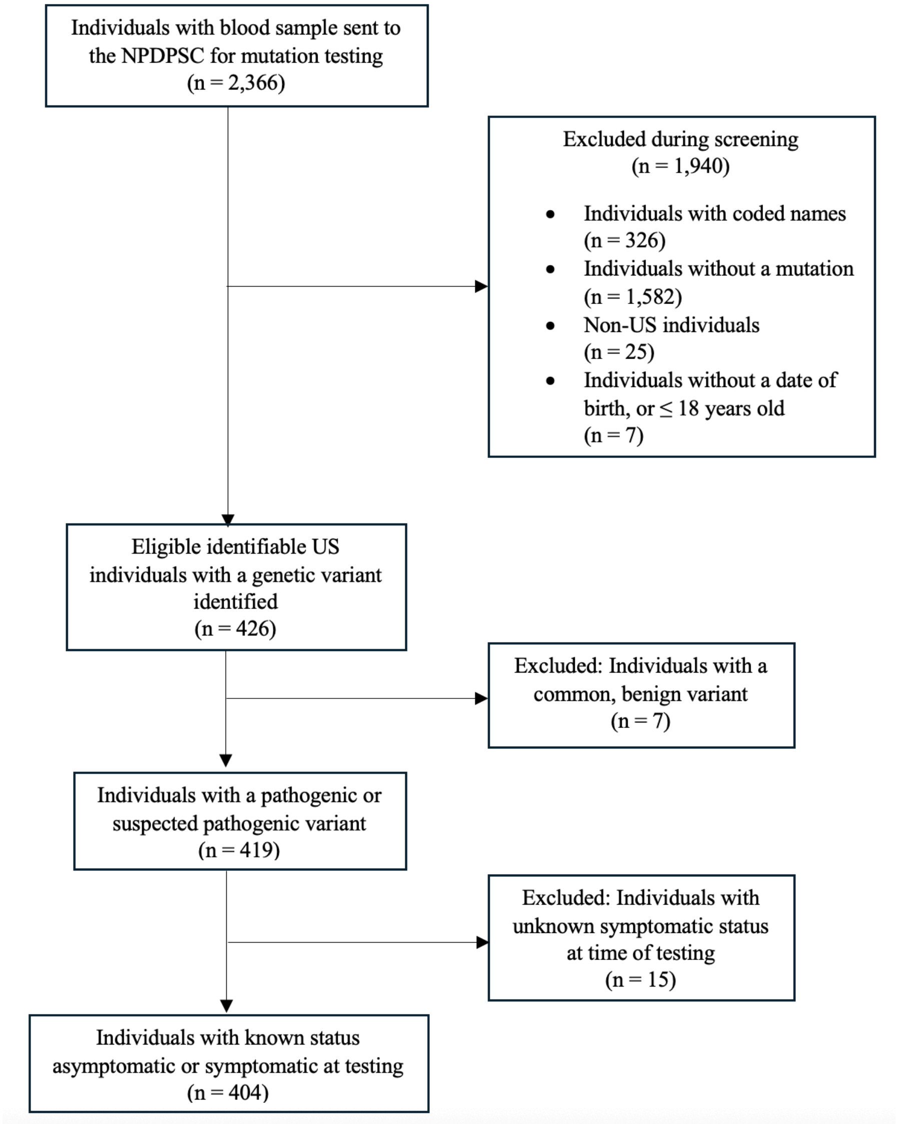

Figure S1. Participant selection flowchart. *This flowchart describes the process of querying and filtering cases from the NPDPSC database for inclusion in this study.*

medRxiv preprint doi: https://doi.org/10.1101/2025.10.03.25337271; this version posted October 9, 2025. The copyright holder for this preprint (which was not certified by peer review) is the author/funder, who has granted medRxiv a license to display the preprint in perpetuity.
It is made available under a CC-BY 4.0 International license .

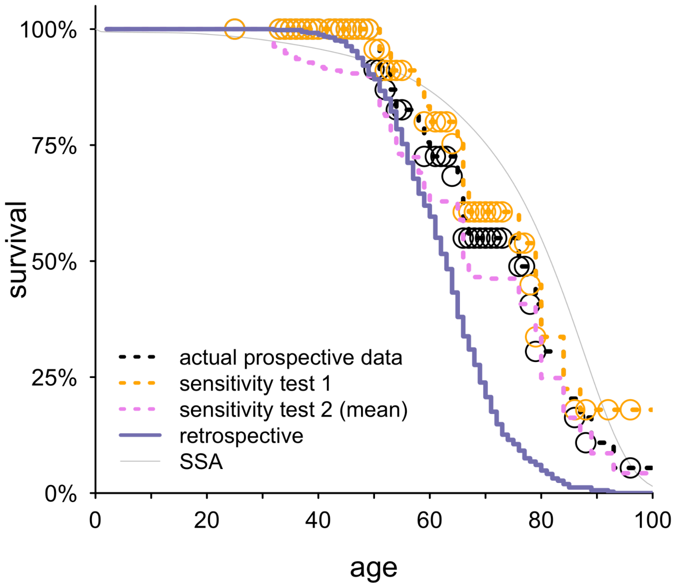

Figure S2. Sensitivity tests for E200K prospective survival curve. *Sensitivity test 1: assume that the 4 E200K deaths identified through public records were not due to prion disease, and are treated as censored. This shifts the prospective survival curve to the right. Sensitivity test 2: assume that N=3 additional E200K deaths occurred and were not detected by public records nor autopsy. The number N=3 was selected because in symptomatic individuals, the lower bound of the 95% confidence interval on sensitivity of autopsy plus public record searches was estimated to be 87.9% (see Results). 20 E200K individuals asymptomatic at test were known to have died, and if this 20 were to represent a sensitivity of 87.9%, then 2.7 additional deaths went undetected, rounded up to 3. In order to test the most extreme possible assumption, in each of 1,000 bootstrap iterations we randomly selected asymptomatic E200K individuals who were age 31 or older at time of testing, and assumed that they died of prion disease in the same year they underwent testing. The age 31 was selected because it is the earliest ever reported disease onset for E200K out of 571 individuals. Sensitivity test 2 represents the most extreme possible assumption because i) we utilized the lower bound of 95% CI of sensitivity rather than the mean estimate, ii) we assumed that deaths occurred immediately after genetic testing, rather than some number of years later, and iii) we assumed that deaths were uniformly distributed from age 31 onward, when in fact deaths at older ages would be far more likely. The thin violet lines show 1,000 individual bootstrap iterations, and the thick dashed blue line shows the mean survival curve across these iterations. The effect of sensitivity test 2 assumptions is to shift the prospective survival curve to the left. Sensitivity tests were then plotted with actual prospective data (black),retrospective survival curve, and SSA actuarial curve.*
